# Effective knockdown of *Drosophila* long non-coding RNAs by CRISPR interference

**DOI:** 10.1093/nar/gkw063

**Published:** 2016-02-04

**Authors:** Sanjay Ghosh, Charlotte Tibbit, Ji-Long Liu

**Affiliations:** MRC Functional Genomics Unit, Department of Physiology, Anatomy and Genetics, University of Oxford, Oxford, OX1 3PT, UK

## Abstract

Long non-coding RNAs (lncRNAs) have emerged as regulators of gene expression across metazoa. Interestingly, some lncRNAs function independently of their transcripts – the transcription of the lncRNA locus itself affects target genes. However, current methods of loss-of-function analysis are insufficient to address the role of lncRNA transcription from the transcript which has impeded analysis of their function. Using the minimal CRISPR interference (CRISPRi) system, we show that coexpression of the catalytically inactive Cas9 (dCas9) and guide RNAs targeting the endogenous *roX* locus in the *Drosophila* cells results in a robust and specific knockdown of *roX1* and *roX2* RNAs, thus eliminating the need for recruiting chromatin modifying proteins for effective gene silencing. Additionally, we find that the human and *Drosophila* codon optimized dCas9 genes are functional and show similar transcription repressive activity. Finally, we demonstrate that the minimal CRISPRi system suppresses *roX* transcription efficiently *in vivo* resulting in loss-of-function phenotype, thus validating the method for the first time in a multicelluar organism. Our analysis expands the genetic toolkit available for interrogating lncRNA function *in situ* and is adaptable for targeting multiple genes across model organisms.

## INTRODUCTION

Recent transcriptome analyses have revealed numerous transcripts that originate from the non-protein-coding part of the genome in metazoa. Long non-coding RNAs (lncRNAs) constitute a subset of these transcripts; they are >200 nt in length, produced by RNA pol II, and undergo RNA processing events and regulate gene expression, in transcript-dependent and -independent manner (1,2). Although linked to several disease conditions including cancers (3), only a fraction has been analyzed in detail and the mechanism of action elucidated. Current strategies to interrogate lncRNA functions include over-expression and knockdown analyses that either targets the endogenous locus (deletion or insertion of genetic elements) or the transcript (RNAi-mediated knockdown and antisense oligonucleotides (ASO)). While the former approach involves alteration of the DNA sequence and/or topology, the latter is dependent on the presence of host machinery; in addition, targeting nuclear RNAs by RNAi or ASO is inefficient. Furthermore, it remains challenging to separate the role of lncRNA transcription from the transcript which makes the interpretation of mutant phenotypes difficult (4).

**Figure 1.**
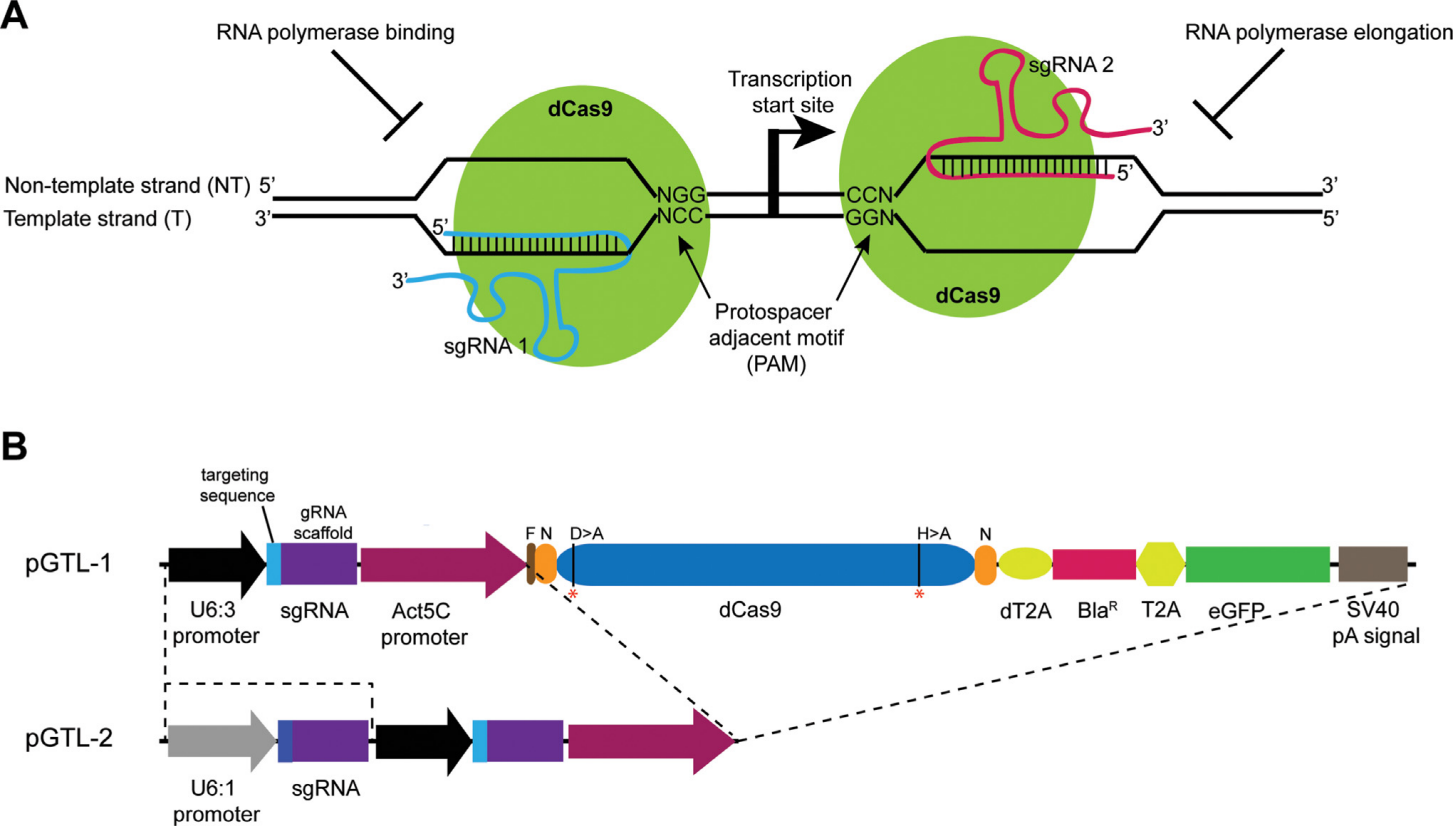
CRISPRi in *Drosophila*. (**A**) Diagram showing the CRISPR interference (CRISPRi) system. To repress transcription, the catalytically inactive Cas9 protein (dCas9, green) is targeted either to the template or non-template DNA strand based on the targeting sequence of the sgRNA and an adjacent PAM sequence. Binding of the dCas9:sgRNA complex upstream of the transcription start site interferes with transcription initiation by preventing recruitment of the RNA polymerase while its assembly at a downstream site prevents transcription elongation. (**B**) Schematic representation of the transfection vectors pGTL-1 and pGTL-2. In pGTL-1, a single guide RNA with a 20 nt targeting sequence and the dCas9 protein are coexpressed under *Drosophila* constitutive promoters *U6:3* and *Actin5C*, respectively. The dCas9 is separated from the blasticidin resistance gene (Bla^R^) and eGFP by self-cleaving T2A peptides (dT2A and T2A). The guide RNA (gRNA) scaffold contains the U6 transcription terminator sequence. The pGTL-2 vector contains an additional sgRNA scaffold under the U6:1 promoter, thus allowing production of two sgRNAs simultaneously with the dCas9 protein. The mutated amino acid residues (D10>A and H841>A) in dCas9 are marked with red asterisks (*). N = NLS sequence, F = FLAG epitope, pA = polyadenylation.

The CRISPR/Cas9 technique had rapidly emerged as a simple, efficient and precise method for DNA modification in the recent years; it is independent of host factors and has been widely used for functional analyses of genomes across the animal kingdom (5,6). It is a two-component system consisting of the Cas9 nuclease which, in complex with a single guide RNA (sgRNA), generates double-stranded breaks. The cleavage specificity is determined by a 20 nt targeting sequence at the 5′ end of the sgRNA and 3 nt protospacer adjacent motif (PAM) sequence abutting the DNA-binding site. In addition to gene editing, modifications of the Cas9 protein and sgRNA have enabled the CRISPR system to be used as a platform for modulating gene expression, thus extending its usefulness. For example, a mutant Cas9 protein that lacks the endonuclease activity (dead Cas9, dCas9) has been used as a RNA-guided DNA-binding complex to program transcription of genes in the endogenous context; fusion with appropriate effector domains results in robust activation (CRISPR activator, CRISPRa) or repression (CRISPR interference, CRISPRi, Figure 1A) of transcription (7–11). The effectiveness of gene silencing by CRISPRi, however, has been variable. While targeting dCas9 to promoter regions of the endogenous genes resulted in substantial reduction in gene function in bacteria (∼ 5-fold, (9)) and yeast (up to 18-fold, (8)), it was inefficient in human cells (∼2.5-fold, (8)). Rather fusion of the transcription repressor KRAB domain to dCas9 protein increased the knockdown efficiency (up to 5-fold in human cells, (8)) presumably due to recruitment of chromatin-modifying factors on the DNA at the targeting site (8). In these studies, however, the knockdown efficiency was determined by protein function and RNA levels were not directly measured. Recently, dCas9-KRAB:sgRNA complex was used to knockdown lncRNAs (upto 7-fold, (7)) in human cells. However, tagging of dCas9 could affect its DNA-binding activity while ectopic assembly of chromatin-modifying complexes could have unintended consequences, especially in regions where genes are located close to each other. Therefore, it is desirable to develop new experimental strategies that would circumvent these limitations and enable functional characterization of lncRNAs and other genes.

In this study, we show that both human and *Drosophila* codon-optimized dCas9 proteins are effective in silencing the lncRNA *roX* in *Drosophila* cells. Furthermore, we observe a robust knock down of the *roX* transcripts *in vivo* resulting in loss-of-function phenotypes, thus validating the effectiveness of the minimal CRISPRi system in *Drosophila*.

## MATERIALS AND METHODS

### Generation of transfection vectors

#### pGTL-1

The Bla^R^ gene was PCR amplified from pCoBLAST (Life Technologies) using primers SG1 and SG2 and ligated between the XbaI and HindIII digested pAc5-STABLE2–neo (12) to replace the eGFP gene, thus generating pSG-Bla-neo. To insert the eGFP gene in place of neo^R^, the pSG-Bla-neo vector was digested with NheI and XhoI and used for ligation with PCR amplified eGFP using primers SG3 and SG4, resulting in pSG-Bla-GFP. The U6:3 cassette containing the promoter and sgRNA sequence was amplified from pCFD3 (13) using SG5 and SG6 primers while the dual promoter U6:1–6:3 cassette was derived from pCFD4 (13) using primers SG7 and SG6. Both PCR products were digested with BglII and ligated into the BglII site of pSG-Bla-GFP vector giving rise to plasmids pSG-Bla-GFP-U6:3 and pSG-Bla-GFP-U6:1–6:3, respectively. The human codon-optimized Cas9 gene together with 3xFLAG and NLS sequences was amplified from pAc5-sgRNA-Cas9-puro (14) and cloned into pUC19 vector. The human Cas9 was mutated by Quikchange II Site-Directed Mutagenesis kit (Stratagene) using primer pairs SG8/SG9 (for D10 > A) and SG10/SG11 (for H841 > A). Next, the human dCas9 cassette (FLAG-NLS-dCas9-NLS) was amplified from the pUC19 clone using primers SG12 and SG13, and used to replace the mCherry gene in pSG-Bla-GFP-U6:3 by KpnI and NotI digestion, resulting in pGTL-1 plasmid.

#### pGTL-2

To construct pGTL-2, the pSG-Bla-GFP-U6:1–6:3 vector backbone and the human dCas9 cassette were amplified using primer pairs SG14/SG15 and SG16/SG17, and used for two-fragment Gibson assembly reaction (New England Biolabs #E2611). The pGTL-2 vector containing the *Drosophila* dCas9 was generated as follows. The *Drosophila* codon-optimized Cas9 containing plasmid was a kind gift of Fillip Port (University of Cambridge) which was used as template to PCR amplify the gene using primers SG18 and SG19, subcloned into pUC19 vector and used for site-directed mutagenesis as described above using the primer pairs SG20/SG21 (for D10 > A) and SG22/SG23 (for H841 > A). The mutated *Drosophila* dCas9 was next amplified using SG24 and SG25 primers and used for Gibson assembly reaction with the pGTL-2 vector backbone amplified using primers SG26/SG27. This replaced the human dCas9 with the *Drosophila* dCas9 which is in-frame with the NLS sequences in the pGTL-2 vector.

#### sgRNA constructs

To minimize potential off-target activity, the targeting sequence predicted to have one target in the *Drosophila* genome was selected using the CRISPRdirect tool (15). The vectors expressing single guide RNA targeting *roX1* were generated using pGTL-1 following the protocol described in the CRISPR fly design website (http://www.crisprflydesign.org/grna-expression-vectors/). Briefly, complementary oligonucleotides containing the 20 nt targeting sequence were phosphorylated, annealed, diluted and ligated to BbsI digested pGTL-1 vector. The oligonucleotide and guide RNA sequences are listed in Supplementary Tables S1 and S2, respectively.

To generate expression vectors containing two sgRNAs, the pGTL-2 plasmids with the human dCas9 and *Drosophila* dCas9 genes were PCR amplified with primers SG28 and SG29. Additionally, oligonucleotides containing the *roX1* and *roX2* targeting sequences were designed (listed in Supplementary Table S1) and used for PCR reaction with pCFD4 plasmid as template (as described for pCFD4 cloning at http://www.crisprflydesign.org/grna-expression-vectors/). The vector PCR fragment was then combined with the sgRNA fragments using the Gibson assembly reaction.

#### pGTL-3

The vector for fly transgenesis was constructed using 2-step Gibson assembly reaction. Four separate PCR reactions were performed, (i) using the pAct5c-Cas9 plasmid ((13), gift of Fillip Port), primers SG42/SG43 were used to generate the vector backbone that lacked the Actin5c:Cas9 cassette, (ii) the *Actin5c* promoter was amplified from pAct5c-Cas9 plasmid using SG44/SG45, (iii) the U6:1-rT8-U6:3-rT9 and U6:1-rTb-U6:3-rTc cassettes were generated from the corresponding pGTL-2 vectors using primers SG46/SG47 and (iv) the *Drosophila* dCas9 gene from pGTL-2 vector by SG48/SG49 primers. Following DpnI digestion, the vector backbone was assembled with the guide RNA cassettes while *Actin5c* with the *Drosophila* dCas9, and subsequently mixed together to generate the transgenesis plasmids. In addition to the *Drosophila* dCas9, the pGTL-3-*roX1* and pGTL-3-*roX2* plasmids support expression of rT8+rT9 and rTb+rTc guide RNAs, respectively.

All plasmid sequences were verified by sequencing.

### Cell culture and transfection

Clone 8 cells (CME W1 cl.8+) were obtained from Drosophila Genomics Resource Center (DGRC) and maintained in M3 media containing 2% Fetal Bovine Serum (FBS) (Sigma #F4135), 5 μg/ml insulin (Sigma #I9278), 2.5% fly extract and penicillin-streptomycin (Life Technologies) in 25°C incubator. Transfection was carried out using Effectene Transfection Reagent (Qiagen) as per manufacturers’ instructions. Cells were seeded in 6-well plates (10^6^cells/well) and incubated with 0.4 μg of plasmid DNA. After 3 days, blasticidin (Life Technologies #R210–01) was added at 25 μg/ml and cells were maintained in the antibiotic thereafter. Surviving cells were inspected for GFP expression and stable cell lines obtained in ∼3 weeks which was used for genomic DNA and RNA preparation.

### RNA preparation and qRT PCR analysis

Total RNA was prepared from cells using the miRNeasy kit (Qiagen) as per manufacturers’ instructions. One microgram of RNA was used for reverse transcription using the QuantiTect Reverse Transcription Kit (Qiagen). For qPCR, the cDNA was diluted 1:10, mixed with primers and SYBRGreen Jumpstart Taq readymix (Sigma) and amplified in Applied Biosystems Fast Real-Time PCR system using primers SG30/SG31 (*roX1*), SG38/SG39 (*roX1 RA*), SG32/SG33 (*roX2*), SG34/SG35 (*rp49*).

The Ct values for *roX* were normalized with *rp49* as the housekeeping gene and ΔΔCt values were calculated. The abundance values were normalized to control cells transfected with pGTL-1 or pGTL-2 producing sgRNAs with a non-targeting sequence (shown as 100%). The data are shown for two independent biological replicates with each replicate containing three technical replicates. The error bar shows the S.E.M. value.

For RT-PCR, total RNA was extracted from clone 8 cells and cDNA prepared as described above. One microliter of 1:10 diluted cDNA was used for PCR amplification using DreamTaq Green PCR master mix (Life Technologies) in a 20 μl volume as per instructions. The primer pairs used for the *roX1* intergenic region (SG36/SG37), *roX1 RA* (SG38/SG39), *roX1 RB* (SG30/SG31) and *rp49* (SG34/SG35) are shown in Supplementary Table S1.

### DNA analysis

For genotyping and analysis of the *roX* loci, the cell lines expressing *Drosophila* dCas9 and sgRNAs (rNT7/rT8 and rTb/rTc) were pelleted and used for genomic DNA preparation using Gel Extraction kit (Qiagen) as described on the webpage of Klaus Forsterman lab (http://www.foerstemann.genzentrum.lmu.de/protocols/). A total of 20 ng of genomic DNA was used for PCR with primers SG31/SG40 (*roX1*) and SG33/SG41 (*roX2*) and the PCR product sequenced.

### Western analysis

One microliter culture of the stable cell lines (∼10^7^cells) were pelleted at 1000 g for 5 mins, suspended in 200 μl of SDS-sample buffer and boiled for 10 min. The proteins in the extract were separated on NuPAGE 4–12% Bis-Tris gels (Life Technologies) according to manufacturers’ instructions, blotted onto PVDF membrane (Amersham Hybond) using Mini Trans-Blot transfer cell (BioRad). The primary antibodies were mouse anti-FLAG antibody (Sigma #F3165) and mouse anti-Tubulin antibody (Sigma #T6199) used at 1:1000 dilution while HRP-conjugated donkey anti-mouse IgG (Jackson Immunoresearch, 1:1000 dilution) was used as the secondary antibody.

### Fly transgenesis

The *Drosophila* transgenesis vector pGTL-3-*roX1* and *-roX2* were injected into *nos-int; attP2* embryos for integration into the attP landing site on chromosome 3 (68A4) at the Microinjection Service of Department of Genetics, University of Cambridge. For coexpression of *roX1* and *roX2* guide RNAs, the GTL-3-*roX1*/TM6cSb and GTL-3-*roX2*/TM6cSb flies were crossed at 29°C and the progeny lacking Sb were scored. The male survival is based on the total number of progeny of the same genotype from the same cross. For comparison, *y,w* parents were kept at 29°C and the progeny counted.

## RESULTS AND DISCUSSION

The *Drosophila* genome encodes numerous lncRNAs which shows tissue- and sex-specific expression (16,17). However, the functions of the majority of these transcripts remain unknown. We reasoned that repression of transcription of the lncRNAs by CRISPRi would overcome the limitations of current methods applied for their analysis and decided to re-evaluate the effectiveness of dCas9 in repressing transcription from endogenous lncRNA loci in *Drosophila* cells. The CRISPR/Cas9 system has been used extensively for gene editing in *Drosophila* (11,13–14,18–26). However, CRISPRi has not been applied till date in *Drosophila*.

To implement CRISPRi in *Drosophila* cells, we first constructed a single transfection vector that allows coexpression of the mutant Cas9 protein and sgRNA, similar to the one we previously described (14). This strategy eliminates the variability between experiments that arise from cotransfection of two plasmids. The transfection vector pGTL-1 (Figure 1B), derived from the multicistronic vector pAc5-STABLE2-neo (12), contains the Bla^R^ gene which allows for efficient selection of the transfected cells and reduces the time to establish stable cell lines (27). In addition, presence of GFP enables easy monitoring of the transformed cells. The human codon-optimized Cas9 gene lacking nuclease activity (dCas9) flanked with NLS sequences and tagged with FLAG epitope at the N-terminal, is expressed under the *Actin5C* promoter. For optimal expression of the guide RNAs, the U6:3–sgRNA cassette was inserted into the vector; this promoter has a higher CRISPR/Cas9-mediated gene editing activity in *Drosophila*, as compared with the commonly used U6:2 promoter (13).

**Figure 2.**
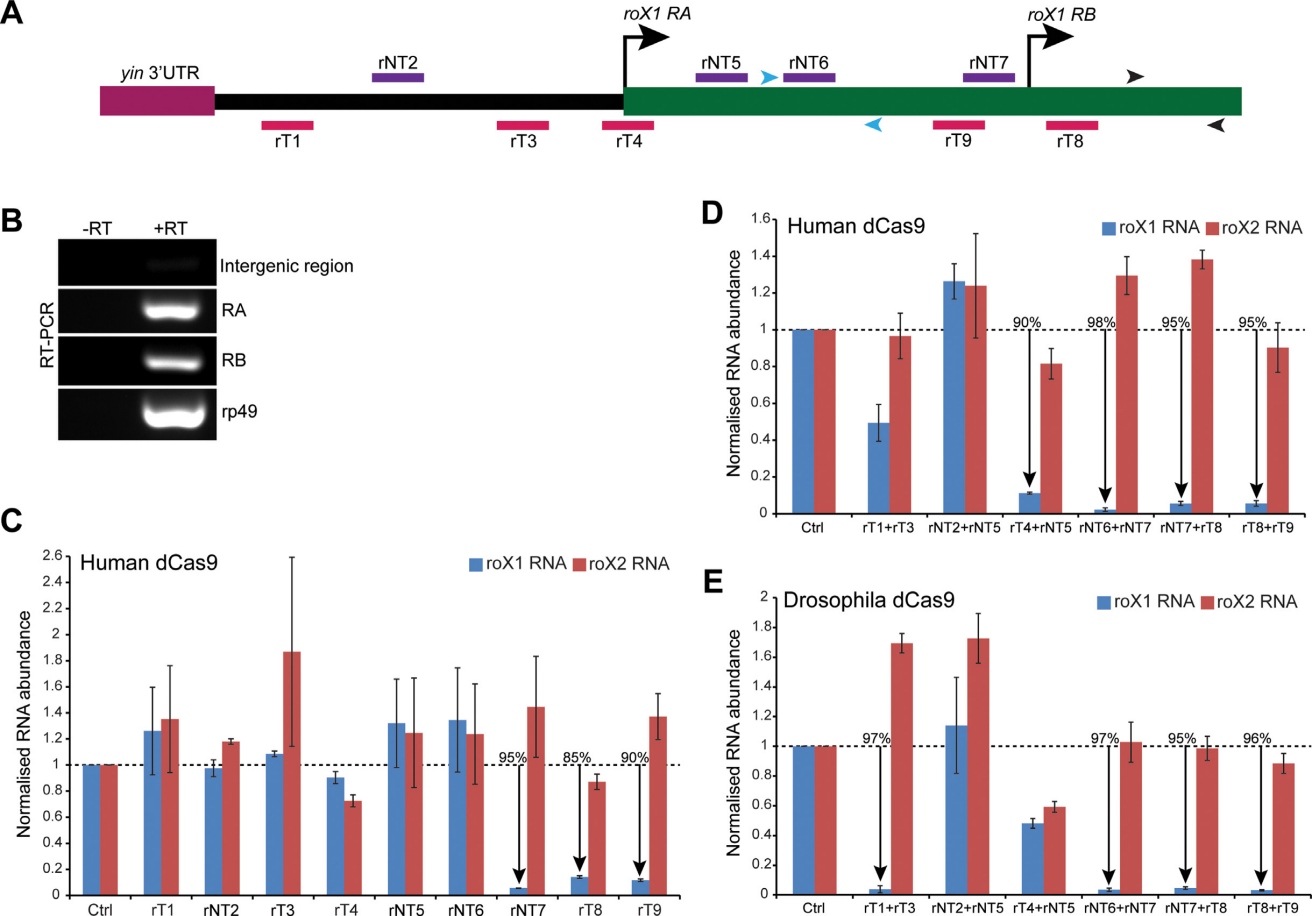
The *roX1* expression is efficiently silenced by CRISPRi. (**A**) Schematic representation of the *Drosophila roX1* locus and the relative positions of the *roX1*-targeting sgRNAs. There are two transcription start sites of *roX1* gene (270 nt apart) which produce isoforms *RA* and *RB* (FlyBase). The sgRNAs targeting the RNA polymerase template (rT, red) and non-template (rNT, violet) strands are shown. The 5′ intergenic region between *roX1 RA* and the upstream gene *yin* is shown in solid black line. The arrowheads mark the position of the primers used to detect *roX1* RNA (black) and *roX1 RA* isoform (blue) for RT-qPCR analysis. (**B**) RT-PCR analysis using clone 8 cells shows the presence of *roX1 RA* and *RB* transcripts. The primers specific to sequences in the intergenic region, *roX1 RA* and *roX1 RB* isoform were used for PCR amplification. (**C, D, E**) RT-qPCR measurement of abundance of the *roX1* and *roX2* transcripts in cell lines coexpressing human (C, D) or *Drosophila* (E) dCas9 protein and sgRNAs (as shown on the x-axis) complementary to the rT or rNT DNA strand at the endogenous *roX1* locus. The cell lines expressing single sgRNA (C), or two guide RNAs simultaneously (D, E) are shown. The gRNAs rNT7, rT8 and rT9 mediate efficient knockdown of the *roX1* transcript, either alone or jointly with others. However, the combinations of rT1+rT3 and rT4+rNT5 which target the *roX1 RA* transcription initiation site, are effective only in pairs in the presence of human dCas9 and *Drosophila* dCas9, respectively. The y-axis shows the enrichment of RNAs relative to *rp49* transcript and normalized to the cells transfected with pGTL-1 containing a non-targeting sequence (Ctrl). The data are shown from two biological replicates, each performed in triplicate. The error bars indicate SEM. The % shows knockdown in percentage as compared with the Ctrl.

**Figure 3.**
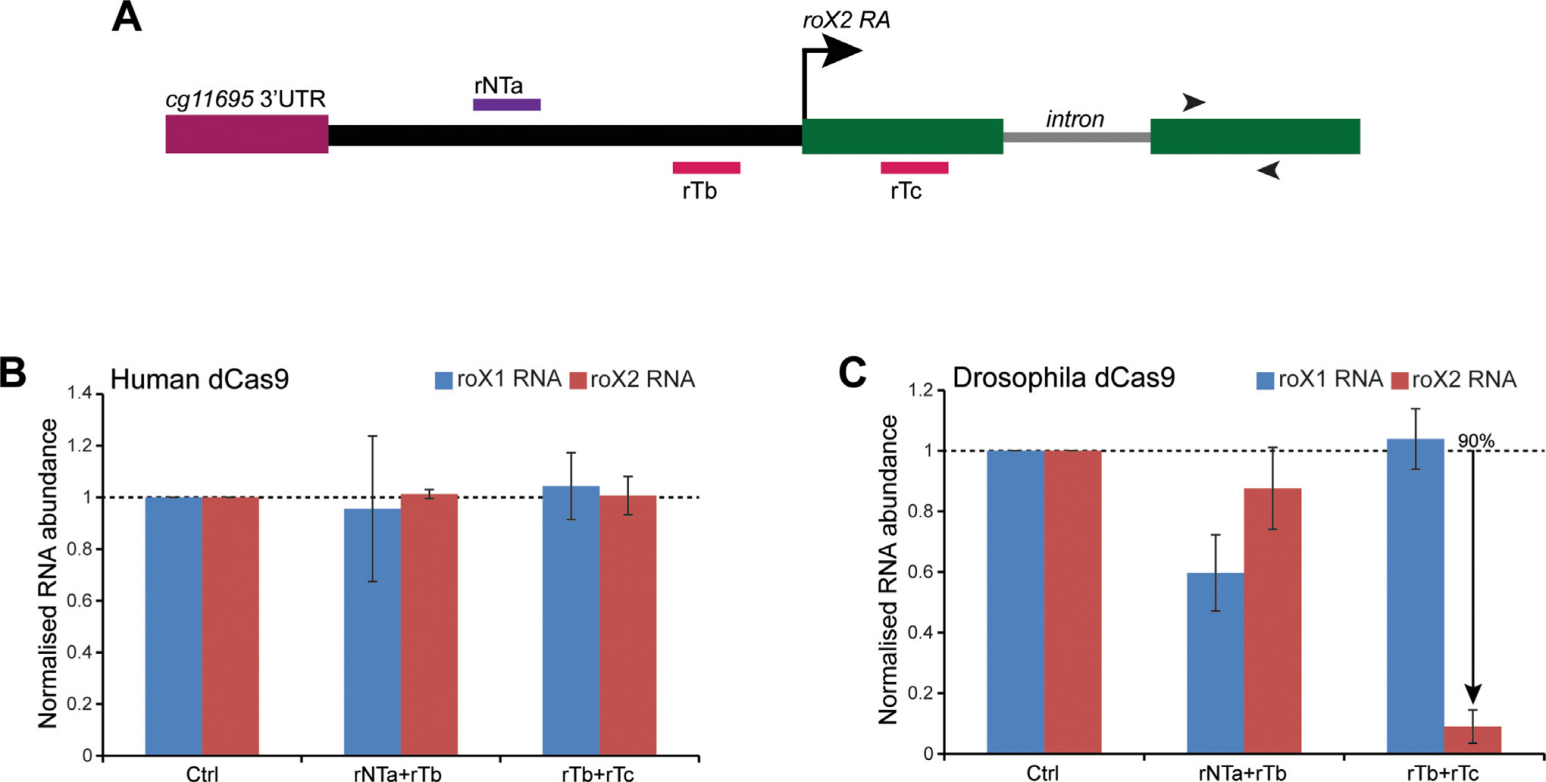
The *roX2* transcript is efficiently down-regulated by CRISPRi. (**A**) Schematic representation of the *Drosophila roX2* locus showing relative positions of the *roX2* targeting sgRNAs. The transcription start site is marked by an arrow while the intron within the *roX2* gene is shown by a solid grey line. The intergenic region between the *roX2* transcription start site and the adjacent gene (*cg11695*) is shown in solid black line. The sgRNAs targeting the rT and rNT strands are shown in red and violet, respectively. The arrowheads mark the position of the primers used to detect *roX2* RNA for RT-qPCR analysis. (**B** and **C**) RT-qPCR measurement of abundance of the *roX1* and *roX2* transcripts in cell lines coexpressing human (B) or *Drosophila* (C) dCas9 protein and two sgRNAs (as shown on the x-axis). While sgRNAs rTb+rTc do not affect *roX1* or *roX2* levels in presence of human dCas9, they specifically knockdown *roX2* transcript levels in cells coexpressing *Drosophila* dCas9. The y-axis shows the enrichment of RNAs relative to *rp49* transcript and normalized to the cells transfected with pGTL-1 containing a non-targeting sequence (Ctrl). The data are shown from two biological replicates, each performed in triplicate. The error bars indicate SEM. The % shows knockdown in percentage as compared with the Ctrl.

As candidate gene, we chose *roX* (RNA on X chromosome), which encodes the lncRNAs involved in dosage compensation in *Drosophila* males (28). The male-specific *roX* RNAs, *roX1* and *roX2*, form complex with the MSL (male-specific lethal) proteins and facilitate targeting of the complex on the X chromosome, necessary for 2-fold hyperactivation of the X-linked genes in males. Genetic analysis shows that *roX1 roX2* double mutants are male lethal which is rescued by either *roX1* or *roX2* cDNA, suggesting functional redundancy in their activity (29,30). Presently, the alleles used for loss-of-function analysis are genomic deletions of the loci or complex chromosomal arrangements. Furthermore, the transcription initiation sites and identity of the regulatory elements remain unclear. We designed multiple sgRNAs targeting the *roX1* and *roX2* locus (Figures 2A and 3A). To determine if effective silencing of *roX* RNAs is dependent on strand-specificity, we chose sequences complementary to both template (rT) and non-template (rNT) strand and in proximity of the predicted transcription start sites. For the assay, we generated stable cell lines using *Drosophila* clone 8 cells which expresses both *roX* RNAs (31).

First, we attempted to suppress transcription of the *roX1* RNA by coexpressing a single guide RNA with the dCas9 protein. The FlyBase (http://flybase.org/) gene model for *roX1* predicts two distinct transcription start sites resulting in a longer *roX1 RA* and a shorter *roX1 RB* isoform. Our data show that both transcripts are present in clone 8 cells (Figure 2B) validating the two initiation sites. Notably, the *roX1 RB* gene region alone was used in the transgenic constructs for rescue analysis (30). Our results show that recruitment of dCas9 adjacent to the *roX1 RB* transcription start resulted in efficient silencing of both *RA* and *RB* isoforms (Figure 2C, Supplementary Figure S1A). Interestingly, sgRNA targeting the non-template (rNT7) and template (rT8) strand showed similar efficacies (95% and 85%, respectively), indicating absence of DNA template bias for silencing as observed in human cells (7). Consistently, rT9, which targets the template strand and partially overlaps with rNT7, shows similar repressive activity. The knockdown is dependent on the *roX1* targeting sequence and is specific as cells with significantly reduced *roX1* levels did not show a concomitant reduction of *roX2* RNA levels. Targeting of dCas9 to regions upstream of the *roX1* transcription start sites (TSS) failed to affect RNA levels. Western analysis showed efficient expression of the dCas9 protein in these cell lines (Supplementary Figure S2A) underlining the critical role of guide RNA sequence in repression. The variability in dCas9 protein expression could be due to chromosomal position effects and/or chromosome copy number changes in this cell line (31). Taken together our results reveal potent sgRNAs for *roX1* silencing and show that assembly of dCas9-sgRNA complex on either strand of DNA can effectively silence lncRNA expression, possibly by preventing the binding and/or elongation of RNA polymerase.

A previous study has shown that the efficiency of CRISPRi could be enhanced by using two sgRNAs targeting the same gene in bacteria (9). To test this in *Drosophila* cells, we inserted the U6:1-sgRNA cassette in the pGTL-1 vector immediately upstream of the U6:3 cassette, thus generating the pGTL-2 vector (Figure 1B); this allows expression of a second sgRNA under the control of U6:1 promoter which also shows a robust activity in *Drosophila* (13). For expression, we chose sgRNA combinations which are located >30 bp apart to allow for efficient binding of the dCas9 protein. As shown in Figure 2D, sgRNAs complementary to the sequences upstream of (rNT6+rNT7) and flanking the *roX1 RB* isoform (rNT7+rT8, rT8+rT9) showed a robust reduction of *roX1* transcript. This is expected and can be attributed to the effectiveness of rNT7, rT8 and rT9 sgRNA as observed previously (Figure 2C), although rNT7 and rT8 are now expressed under the control of U6:1 promoter. Remarkably, we observe an effective silencing of *roX1* RNA by sgRNAs targeting the *roX1 RA* transcription region. Although ineffective individually, the sgRNA combinations targeting the template strand (rT1+rT3) or both the template and non-template strand (rT4+rNT5) show striking loss in *roX1* RNA levels (50% and 90%, respectively). We did not observe a strong correlation between the dCas9 levels and transcription inhibition (Supplementary Figure S2B). Collectively, these results suggest that binding of multiple dCas9 protein at the transcriptional start sites can augment the effectiveness of transcriptional silencing.

We next determined whether codon optimization of dCas9 protein could enhance the combinatorial activity of CRISPRi. The *Drosophila* codon-optimized Cas9 has been used recently for *in vivo* gene editing; however, expression of the protein with strong, ubiquitous Gal4 drivers led to substantial lethality for yet unknown reasons (13). We generated catalytically inactive *Drosophila* Cas9 by mutating the amino acids essential for nuclease activity (D10A and H841A) and substituted the human dCas9 in pGTL-2 vector. As shown in Figure 2E, coexpression of sgRNAs and *Drosophila* dCas9 recapitulates silencing activity of the sgRNAs to a similar extent as observed with the human dCas9 protein (rNT6+rNT7, rNT7+rT8 and rT8+rT9). Western analysis showed efficient translation of *Drosophila* dCas9 protein (Supplementary Figure S2B). The cell lines did not show any overt phenotype suggesting that *Drosophila* dCas9 expression is not toxic and the protein is active in silencing gene expression. However, as compared with human dCas9, we observed a striking reduction (97% for *Drosophila* dCas9 versus 50% for human dCas9) of *roX1 RA* and *RB* RNA with sgRNAs that target the 5′ region of *roX1 RA* TSS (rT1+rT3) (Figure 2E, Supplementary Figure S1B). The enhanced effects of this guide RNA combination, although ineffective individually, could be due to targeting of redundant regulatory elements in the *roX1* intergenic region. We also noted that cells expressing rT4+rNT5 show ∼40% reduction in *roX2* RNA levels (Figure 2E) – this is unlikely due to an off-target effect as Gapdh and CTPsyn RNA levels are not affected similarly (Supplementary Figure S3A). Importantly, DNA sequencing revealed that the silencing effect of the *Drosophila* dCas9-sgRNA complex at the endogenous loci does not involve changes in the DNA sequence (Supplementary Figure S4).

Since *roX1* targeting sgRNAs showed a combinatorial effect in gene silencing and were potent in presence of *Drosophila* dCas9, we tested whether a similar strategy could be used to abrogate *roX2* transcription. For analysis, we chose three targeting sequences around the *roX2 RA* TSS, complementary to both the non-template (rNTa) and template (rTb and rTc) strands (Figure 3A). Coexpression of the sgRNAs with human dCas9 did not affect the *roX2* transcript level significantly (Figure 3B). However, when the same combinations of sgRNAs were expressed together with the *Drosophila* dCas9, we observed a significant reduction (90% knockdown) of *roX2* transcript in the sgRNAs flanking the transcription start site (rTb+rTc, Figure 3C). Importantly, the *roX1* transcript was unaffected in the same cell line, demonstrating the specificity of the knockdown. Consistently, Gapdh and CTPsyn RNA levels also remain unaltered (Supplementary Figure S3B). Notably, the dCas9 protein levels did not correspond to the degree of suppression (Supplementary Figure S2C). These data, together with our previous observation with *roX1*, supports the enhanced activity of *Drosophila* dCas9 in mediating transcription repression via CRISPRi.

**Figure 4.**
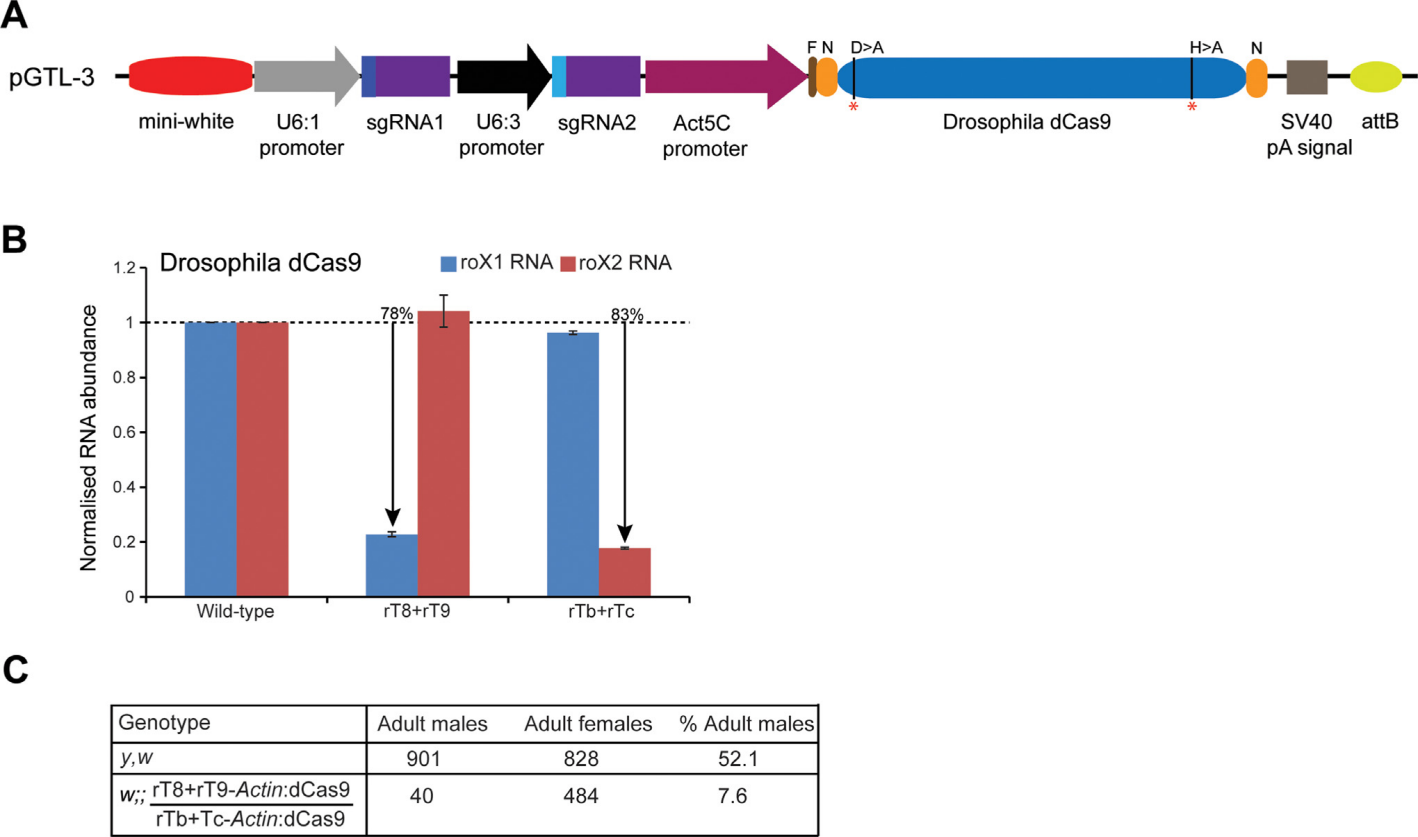
Knock down of *roX1* and *roX2* RNAs *in vivo*. (**A**) Schematic representation of the *Drosophila* transgenesis vectors pGTL-3. The dominant selection marker *mini-white* is followed by the dual guide RNA expressing cassette under the control of *U6:1* and *U6:3* promoters while the expression of *Drosophila* dCas9 is controlled by the constitutively active promoter *Actin5c*. The mutated amino acid residues (D10 > A and H841 > A) in dCas9 are marked with red asterisks (*). N = NLS sequence, F = FLAG epitope, pA = polyadenylation. (**B**) RT-qPCR analysis showing robust depletion of *roX1* and *roX2* RNAs in the males of fly lines expressing the corresponding guide RNAs (shown on the x-axis). The y-axis shows the enrichment of RNAs relative to *rp49* transcript and normalized to the wild-type (*y,w*) males. The data are shown from two biological replicates, each performed in triplicate. The error bars indicate SEM. The % shows knockdown in percentage as compared with the wild-type. (**C**) Table showing a severe reduction in the male progeny simultaneously expressing *roX1* and *roX2* guide RNAs (rT8+rT9-Act:dCas9/rTb+rTc-Act:dCas9). The percentage male survival is calculated based on the total number of progeny of the same genotype recovered in the same cross. The *y,w* strain was used as wild-type.

Till date, CRISPRi has not been applied in any multicellular organism. To determine the effectiveness of CRISPRi in repressing transcription of *roX* RNAs *in vivo*, we generated two transgenic flies expressing *Drosophila* dCas9 and sgRNAs under constitutively active promoters. For analysis, we chose the guide RNAs that showed potent activity in downregulating the corresponding *roX* genes in the *Drosophila* cell line – rT8+rT9 to target *roX1* and rTb+rTc for *roX2*. For transgenesis, we modified the *pAct5c*-Cas9 vector (13) to generate pGTL-3 vector – the Cas9 gene was replaced by *Drosophila* dCas9 and the U6:1-sgRNA-U6:3-sgRNA cassettes were inserted between the *mini-white* gene and the *Act5c* promoter (Figure 4A). This vector allows for stable site-specific integration into the *Drosophila* genome, thus eliminating position-effect and variability in transgene expression. Indeed, the dCas9-*roX1* and dCas9-*roX2* fly lines efficiently express similar levels of the *Drosophila* protein (Supplementary Figure S2D) and were viable indicating that expression of these transgenes was not toxic. RT-qPCR showed a robust and specific reduction in the *roX1* and *roX2* RNA levels (Figure 4B) which is consistent with our analyses in the cell line and, thus, validates the repressive activity of *Drosophila* dCas9 *in vivo*. The knock down is likely to be an underestimation as *Actin5c* is not expressed in some adult tissues (32) and is excluded from the germline. Remarkably, as compared with females, we observed a drastic reduction in the viability of males coexpressing *roX1* and *roX2* guide RNAs (7.6% dCas9-*roX1*/dCas9-*roX2* males versus 52.1% wild-type males, Figure 4C). The escaper males are fertile and show no obvious phenotypic defects. This is consistent with a strong loss-of-function of *roX* (33). Thus, we conclude that recruitment of the *Drosophila* dCas9 at *roX1* and *roX2* locus disrupts their expression by suppressing transcription, resulting in male lethality.

In this report, we describe an effective loss-of-function approach for studying lncRNA genes in *Drosophila* by a minimal CRISPRi system that does not require fusion of effector domains to dCas9. Using this straightforward method, we achieved high levels of knock down of the *roX* RNAs (up to 50-fold) by targeting the endogenous loci in *Drosophila* cells. Our strategy overcomes the previous limitations of genetic approaches used for assessing lncRNA function – it does not introduce changes in the DNA and eliminates ectopic recruitment of chromatin silencing complexes. Our results demonstrate that targeting multiple sgRNAs that flank the transcription start site of genes improves the knock down potency of CRISPRi. In this regard, recent development of engineered Cas9 proteins with flexible PAM recognition properties and enhanced specificity should increase the choice of targetable sequences while minimizing off-target effects (34,35). Since the guide RNA activity remains difficult to predict and characterization of promoter and transcription regulatory elements of lncRNAs remain incomplete, we recommend testing multiple guide RNAs to select potent guides for analysis. The design of our transfection vector and cell-based assay allows quick evaluation of the effectiveness of several sgRNAs before their use in *in vivo* studies, ranging from scaling to silencing of gene expression as well as promoter and/or enhancer mapping experiments. This strategy, in combination with traditional knock down approaches, should allow functional and mechanistic exploration of lncRNAs across metazoa, thus shedding light on these enigmatic transcripts that constitute the ‘dark matter’ of the genome.

## Supplementary Material

SUPPLEMENTARY DATA
