# Supplementary material for: Effective knockdown of Drosophila long non-coding RNAs by CRISPR interference
Source: Nucleic Acids Res. 2016 Feb 4;44(9):e84. doi: 10.1093/nar/gkw063 (PMC4872081; doi:10.1093/nar/gkw063)
Supplement: SUPPLEMENTARY DATA [file supp_44_9_e84__index.html]

Effective knockdown of Drosophila long non-coding RNAs by CRISPR interference — Effective knockdown of Drosophila long non-coding RNAs by CRISPR interference — SUPPLEMENTARY DATA 

# Effective knockdown of *Drosophila* long non-coding RNAs by CRISPR interference

## SUPPLEMENTARY DATA

- SUPPLEMENTARY DATA
