# Supplementary material for: Effective knockdown of Drosophila long non-coding RNAs by CRISPR interference
Source: Nucleic Acids Res. 2016 Feb 4;44(9):e84. doi: 10.1093/nar/gkw063 (PMC4872081; doi:10.1093/nar/gkw063)
Supplement: SUPPLEMENTARY DATA [file supp_gkw063_nar-00007-met-h-2016-File006.pdf]

## Supplementary Data

# Effective knockdown of *Drosophila* long noncoding RNAs by CRISPR interference

Sanjay Ghosh*, Charlotte Tibbit and Ji-Long Liu*

*MRC Functional Genomics Unit, Department of Physiology, Anatomy and Genetics, University of Oxford, Oxford OX1 3PT, United Kingdom.*

* To whom correspondence should be addressed.

Email: jilong.liu@dpag.ox.ac.uk, Tel: +44 1865 285833;

Correspondence may also be addressed to sanjay.ghosh@dpag.ox.ac.uk

**Supplementary Figure S1. The *roX1* isoforms are affected similarly by CRISPRi.**

RT-qPCR analysis of the cell lines that express either single (A) or two guide RNAs (B) targeting *roX1* locus using primers specific for the *RA* isoform. Suppressing transcription from the *roX1 RA* (rT1+rT3) or *RB* (rTN7 and rNT7+rT8) transcription start site shows a strong decrease in the levels of both the isoforms. Notably, the guide RNAs ineffective in CRISPRi does not affect *roX1 RA* and *RB* abundance considerably. The y-axis shows the enrichment of RNAs relative to *rp49* transcript and normalised to the cells transfected with pGTL-1 (A) or pGTL-2 (B) with a non-targeting sequence (Ctrl). (A) The data shows the mean values from experiment performed in triplicate and the data in (B) is from two biological replicates each performed in triplicate. The error bars show SEM.

**Supplementary Figure S2. Expression of dCas9 proteins in the *Drosophila* cell lines and *in vivo*.**

Western blot analysis showing expression of human and *Drosophila* codon-optimised dCas9 proteins in the cell lines (A-C) and the transgenic fly lines (D). The blots were probed with anti-FLAG antibody to detect the FLAG-tagged dCas9 protein while the loading control protein Tubulin was detected by its corresponding antibody. Dm dCas9 in (D) refers to *Drosophila* dCas9 protein. Absence of signal in cells that do not express dCas9 (A, lane 1) and wild-type flies (D, lane 1) demonstrates specificity of anti-FLAG staining.

**Supplementary Figure S3. Knock down of *roX1* and *roX2* transcripts do not affect CTPsynthase and Gapdh RNA levels.**

RT-qPCR analysis shows that the cells co-expressing *Drosophila* dCas9 and *roX1* (A) or *roX2* (B) targeting guide RNAs (shown on the x-axis) do not cause concomitant reduction of CTP synthase (CTPsyn) and Gapdh RNA levels as compared with the targeted loci. The y-

axis shows the enrichment of RNAs relative to *rp49* transcript and normalised to the cells transfected with pGTL-2 with a non-targeting sequence (Ctrl). The data shows the mean values from experiment performed in triplicate.

**Supplementary Figure S4. The *roX1* and *roX2* loci are not affected by dCas9-sgRNA targeting.**

(A, B) DNA sequencing chromatograms shows that the sequences around the *roX1* (A) and *roX2* (B) loci targeted by *Drosophila* dCas9:sgRNA complexes that cause efficient knockdown of the corresponding transcripts, are unchanged. For comparison, the sequence from the control (Ctrl) cell line is shown in the upper panels of A and B. The sgRNA targeting sequences are shown in solid black lines while the transcription start sites are marked by a dashed line with an arrow.

**Supplementary Table 1**

| Primer | Sequence (5’>3’) |
|---|---|
| SG1 | ACGCTCTAGAATGGCCAAGCCTTTGTCTC |
| SG2 | ACGAAGCTTGCCCTCCCACACATAACC |
| SG3 | ACGCGCTAGCATGGTGAGCAAGGGCGAG |
| SG4 | CAGGCCTCGAGGTACAGCTCGTCCATGCCG |
| SG5 | CAGAGATCTCTTTTTTGCTCACCTGTGATTG |
| SG6 | CAGAGATCTGGCACACCACAAATATACTGTTG |
| SG7 | CAGAGATCTATTTTCAACGTCCTCGATAGTATAG |
| SG8 | AAGTACAGCATCGGCCTGGCCATCGGCACCAACTCTGTG |
| SG9 | CACAGAGTTGGTGCCGATGGCCAGGCCGATGCTGTACTT |
| SG10 | TCCGACTACGATGTGGACGCCATCGTGCCTCAGAGCTTTC |
| SG11 | GAAAGCTCTGAGGCACGATGGCGTCCACATCGTAGTCGGA |
| SG12 | CATGGTACCATGGACTATAAGGACCAC |
| SG13 | CATGGCGGCCGCCGCCTGATCCCTTTTTCTTTTTTG |
| SG14 | GGTGGCGAATTCCACCAC |
| SG15 | CTTTTTCTTTTTTGCCTGGCC |
| SG16 | TACTAGTCCAGTGTGGTGGAATTCGCCACCATGGACTATAAGGACCACGACG |
| SG17 | GGCCGGCCAGGCAAAAAAGAAAAAGGGATCAGGCGGCGGCCGCGAAGGACGCGGCAG |
| SG18 | CCAGGAATTCATGGATAAGAAGTATAGCATCGGC |
| SG19 | CTGAAGCTTCTCCTGCTTGCGCTTCTC |
| SG20 | GAAGTATAGCATCGGCCTGGCCATCGGCACCAACAGCG |
| SG21 | CGCTGTTGGTGCCGATGGCCAGGCCGATGCTATACTTC |
| SG22 | GTCCGACTACGACGTCGATGCCATTGTGCCCCAGTCGTTC |
| SG23 | GAACGACTGGGGCACAATGGCATCGACGTCGTAGTCGGAC |
| SG24 | CGGTATCCACGGAGTCCCAGCAGCCATGGATAAGAAGTATAGCATCGGC |
| SG25 | ATCGCCGCCCAACTGGG |
| SG26 | GGCTGCTGGGACTCCGTG |
| SG27 | CGACCTGTCCCAGTTGGGCGGCGATAAAAGGCCGGCGGCCAC |
| SG28 | GAAGTTCACCCGGATATCTTTCCTATATA |
| SG29 | GTTTTAGAGCTAGAAATAGCAAGTTAAAATAAG |
| SG30 | CGAAATCTCTGGCAAGATGTAG |
| SG31 | CCACAGGCACGTTTTCTG |
| SG32 | CGAAGCAAAATCAAGCAAG |
| SG33 | GTCAACCATGAAAACAATTCG |
| SG34 | GCTAAGCTGTCGCACAAA |
| SG35 | GAACTTCTTGAATCCGGTG |
| SG36 | TGTGTCCCAAACATTGCTG |
| SG37 | CAAGATACAGTTCACAGCGAAC |
| SG38 | CGTTTAGGCCAGTTGGTAAG |
| SG39 | GAAACTACGCCCATGTCTTC |
| SG40 | TCCATACCCATATCGACAGC |

| | |
|---|---|
| SG41 | ACTGCGCCAAGGAGTTC |
| SG42 | AAAAGGCCGGCCAGGCAAAAAAGAAAAAGTGACTCGAG GGTACCTCTAGAGG |
| SG43 | CGAAGTTATGCTAGCCTAGTTCCAG |
| SG44 | TTGTGCTCGGCAACAGTATATTTGTGGTGTCTAGCGGATC CAAGCTTTGC |
| SG45 | CGGGGATCGATCCTGTAAG |
| SG46 | ACTGGAACTAGGCTAGCATAACTTCGATTTTCAACGTCCT CGATAGTATAGTG |
| SG47 | ACACCACAAATATACTGTTGCCG |
| SG48 | CCATTGCAGCTTACAGGATCGATCCCCGGGTACCATGGAC TATAAGGACCAC |
| SG49 | CTTTTTCTTTTTTGCCTGGC |
| rT1 Fwd | GTCGATACAATAATATTAGCTAAC |
| rT1 Rvs | AAACGTTAGCTAATATTATTGTAT |
| rNT2 Fwd | GTCGTAACATTAACAGCAATGTTT |
| rNT2 Rvs | AAACAAACATTGCTGTTAATGTTA |
| rT3 Fwd | GTCGCTCGTTGGAAAAAGTTACTG |
| rT3 Rvs | AAACCAGTAACTTTTTCCAACGAG |
| rT4 Fwd | GTCGTAGAACAATTACGTTCGGAG |
| rT4 Rvs | AAACCTCCGAACGTAATTGTTCTA |
| rNT5 Fwd | GTCGTACTATTACCGATCGATCAC |
| rNT5 Rvs | AAACGTGATCGATCGGTAATAGTA |
| rNT6 Fwd | GTCGTGTTAATTTGCCTTACCAAC |
| rNT6 Rvs | AAACGTTGGTAAGGCAAATTAACA |
| rNT7 Fwd | GTCGCGAAAAAACGAGGGCCATTA |
| rNT7 Rvs | AAACTAATGGCCCTCGTTTTTTCG |
| rT8 Fwd | GTCGAAGAAAAGTGTTAGTTACC |
| rT8 Rvs | AAACGGTAACTAACACTTTTCTT |
| rT9 Fwd | GTCGTCGACAAGTGGCAGCCCTAA |
| rT9 Rvs | AAACTTAGGGCTGCCACTTGTCGA |
| rT1+rT3 Fwd | TATATAGGAAAGATATCCGGGTGAACTTCGATACAATAA TATTAGCTAACGTTTTAGAGCTAGAAATAGCAAG |
| rT1+rT3 Rvs | ATTTTAACTTGCTATTTCTAGCTCTAAAACCAGTAACTTTT TCCAACGAGCGACGTTAAATTGAAAATAGGTC |
| rNT2+rNT5 Fwd | TATATAGGAAAGATATCCGGGTGAACTTCGTAACATTAA CAGCAATGTTTGTTTTAGAGCTAGAAATAGCAAG |
| rNT2+rNT5 Rvs | ATTTTAACTTGCTATTTCTAGCTCTAAAACGTGATCGATC GGTAATAGTACGACGTTAAATTGAAAATAGGTC |
| rT4+rNT5 Fwd | TATATAGGAAAGATATCCGGGTGAACTTCGTAGAACAAT TACGTTCGGAGGTTTTAGAGCTAGAAATAGCAAG |
| rNT6+rNT7 Fwd | TATATAGGAAAGATATCCGGGTGAACTTCGTGTTAATTTG CCTTACCAACGTTTTAGAGCTAGAAATAGCAAG |
| rNT6+rNT7 Rvs | ATTTTAACTTGCTATTTCTAGCTCTAAAACTAATGGCCCTC GTTTTTTCGCGACGTTAAATTGAAAATAGGTC |
| rNT7+rT8 Fwd | TATATAGGAAAGATATCCGGGTGAACTTCGCGAAAAAAC GAGGGCCATTAGTTTTAGAGCTAGAAATAGCAAG |
| rNT7+rT8 Rvs | ATTTTAACTTGCTATTTCTAGCTCTAAAACGGTAACTAAC ACTTTTCTTCGACGTTAAATTGAAAATAGGTC |

| | |
|---|---|
| rNTa+rTb Fwd | TATATAGGAAAGATATCCGGGTGAACTTCGCTGCATGAA TCCGAAAATAGGTTTTAGAGCTAGAAATAGCAAG |
| rNTa+rTb Rvs | ATTTTAACTTGCTATTTCTAGCTCTAAAACTTGATATTTTC GAGTTTTGGCGACGTTAAATTGAAAATAGGTC |
| rTb+rTc Fwd | TATATAGGAAAGATATCCGGGTGAACTTCGCCAAAACTC GAAAATATCAAGTTTTAGAGCTAGAAATAGCAAG |
| rTb+rTc Rvs | ATTTTAACTTGCTATTTCTAGCTCTAAAACTAGCTTAGTGT GACCAGGCCGACGTTAAATTGAAAATAGGTC |

**Supplementary Table 2**

| gRNA | Targeting sequence | PAM |
|---|---|---|
| rT1 | ATACAATAATATTAGCTAAC | TGG |
| rNT2 | TAACATTAACAGCAATGTTT | GGG |
| rT3 | CTCGTTGGAAAAAGTTACTG | TGG |
| rT4 | TAGAACAATTACGTTCGGAG | TGG |
| rNT5 | TACTATTACCGATCGATCAC | TGG |
| rNT6 | TGTTAATTTGCCTTACCAAC | TGG |
| rNT7 | CGAAAAAACGAGGGCCATTA | GGG |
| rT8 | AAGAAAAGTGTTAGTTACC | AGG |
| rT9 | TCGACAAGTGGCAGCCCTAA | TGG |
| rNTa | CTGCATGAATCCGAAAATAG | CGG |
| rTb | CCAAAACTCGAAAATATCAA | GGG |
| rTc | GGCCTGGTCACACTAAGCTA | GGG |

A

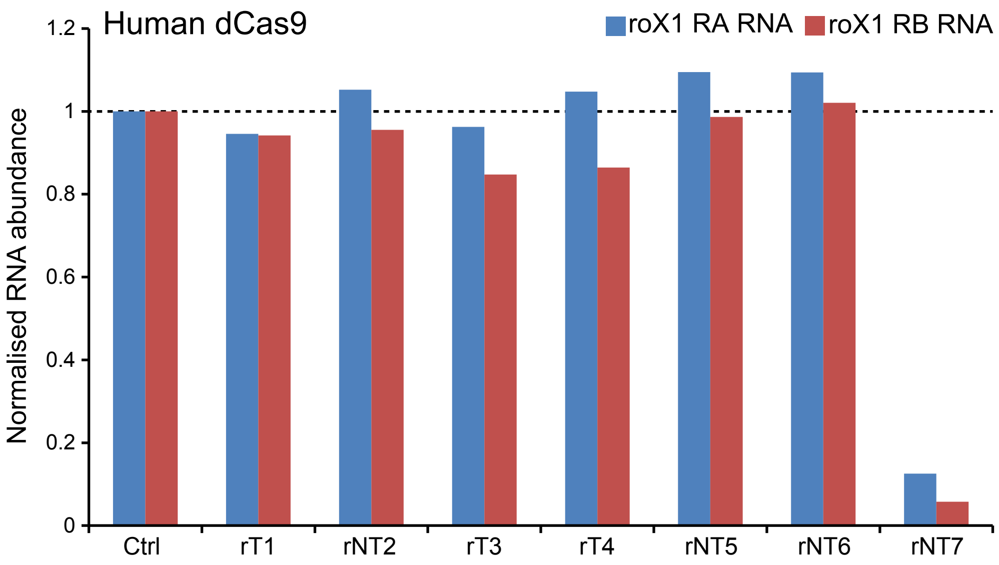


B

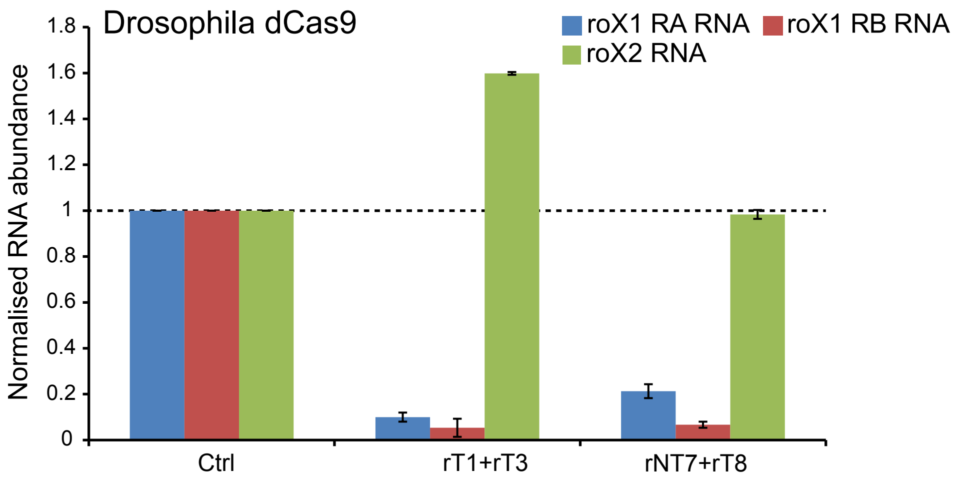


**Supplementary Figure S1** **Ghosh et al.**

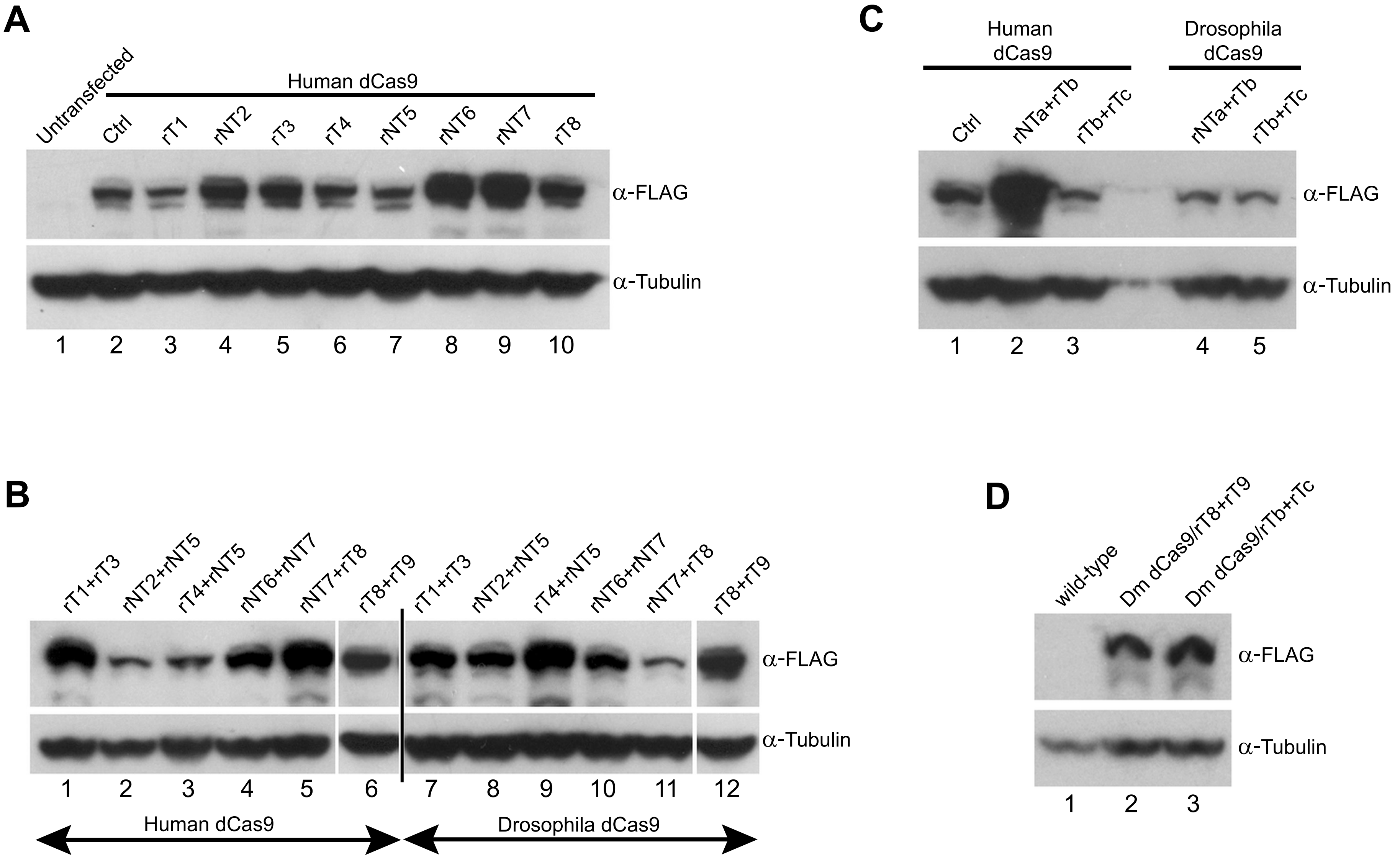


**Supplementary Figure S2**

**Ghosh et al.**

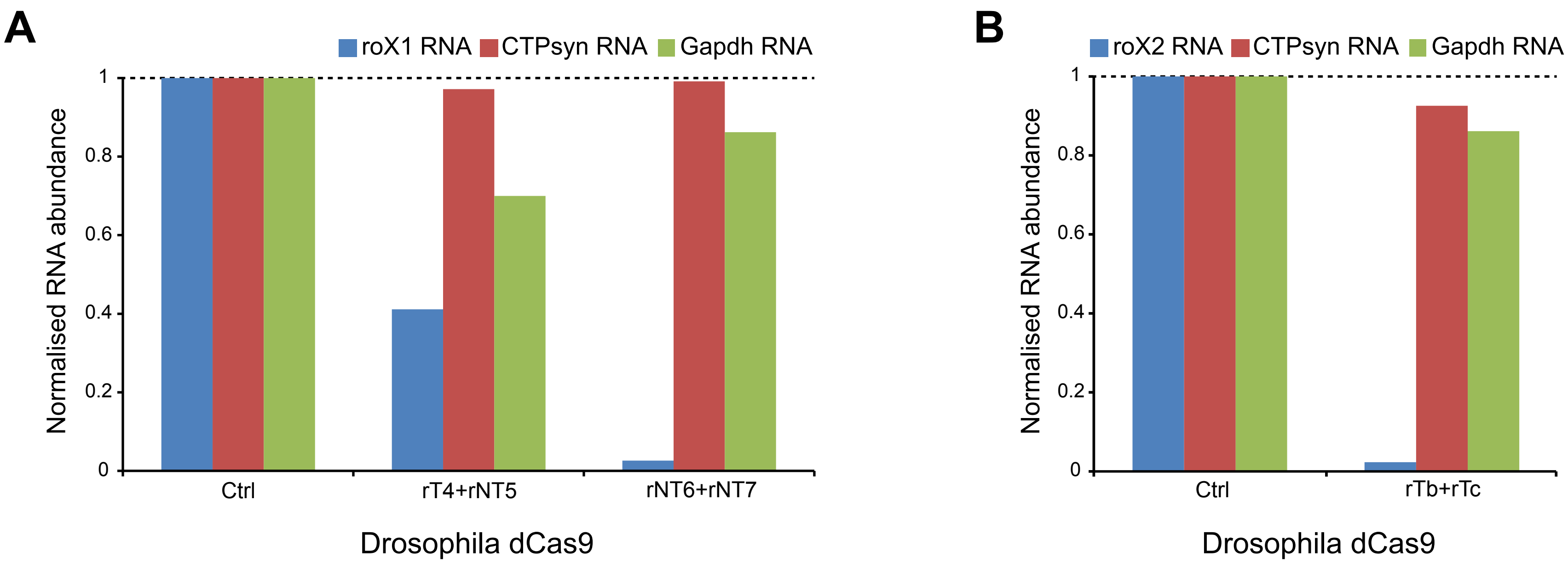


**Supplementary Figure S3** **Ghosh et al.**

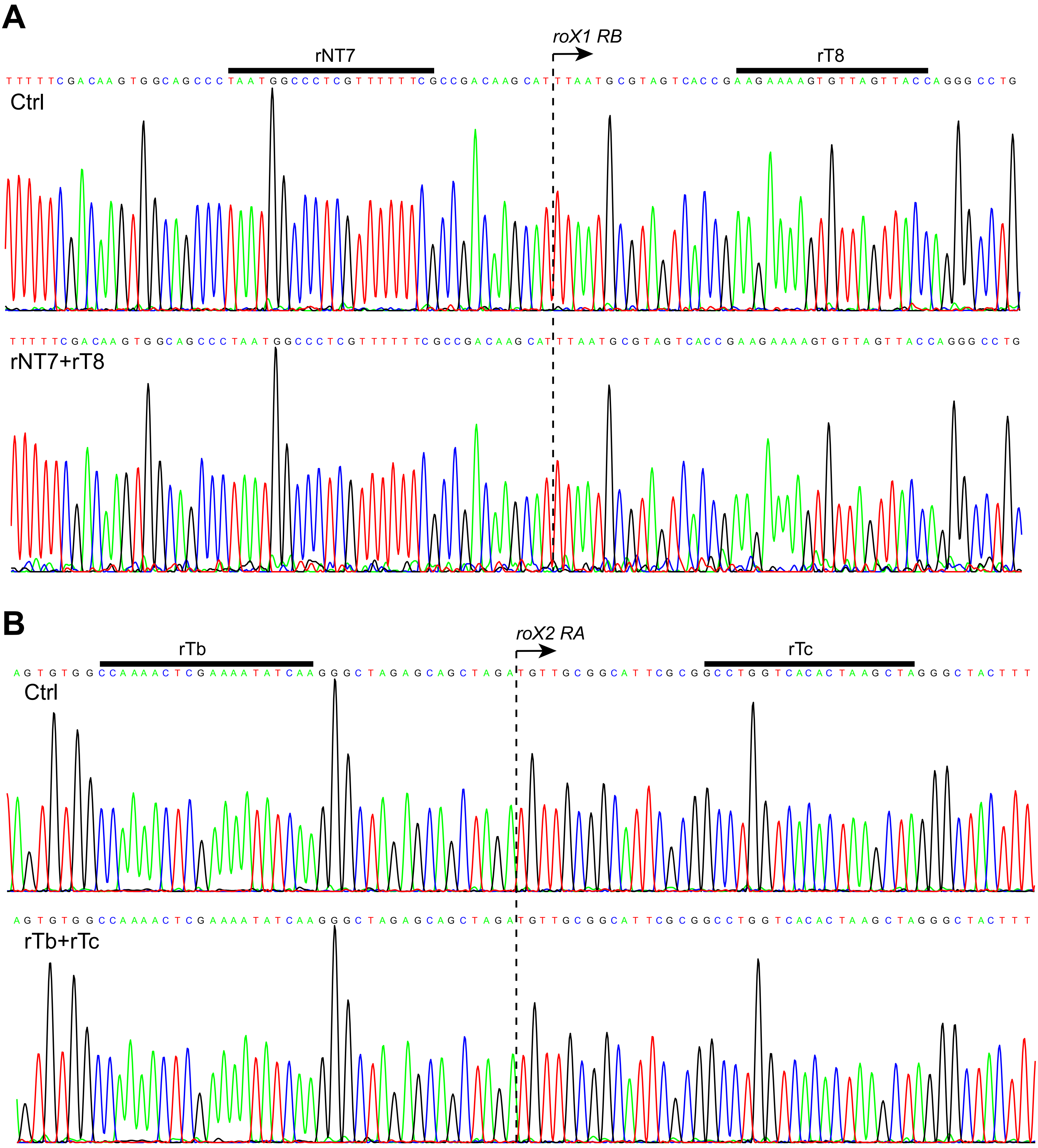


**Supplementary Figure S4** **Ghosh et al.**
